# Technique-dependent differences in internal adaptation, porosity and degree of conversion of short fibre-reinforced composite restorations in deep MOD cavities

**DOI:** 10.1007/s00784-026-07046-9

**Published:** 2026-07-27

**Authors:** András Gábor Jakab, Alíz Alföldi, Zsuzsanna Őri, Tamás Kiss, Kata Bankó, Viktória Néma, Sándor Kunsági-Máté, József Szalma, Márk Fráter, Edina Lempel

**Affiliations:** 1 Doctoral School of Clinical Medicine, Albert Szent-Györgyi Medical School, University of Szeged, Korányi Fasor 6, Szeged, 6720 Hungary; 2 Food and Nutrition Analytical Laboratory, Institute of Basic Health Sciences and Analytical Laboratory Research, Faculty of Health Sciences, University of Pécs, Vörösmarty Street 4, Pécs, 7621 Hungary; 3 Department of Physical Chemistry and Materials Science, Institute of Chemistry, Faculty of Sciences, University of Pécs, Ifjúság Street 6, Pécs, 7624 Hungary; 4 Department of Pharmacology and Pharmacotherapy, University of Pécs Medical School, Szigeti street 12, Pécs, 7624 Hungary; 5 Department of Restorative Dentistry and Periodontology, University of Pécs Medical School, Tüzér Street 1, Pécs, 7623 Hungary; 6 Department of Operative and Esthetic Dentistry, Faculty of Dentistry, University of Szeged, Tisza Lajos Blvd 64-66, Szeged, 6720 Hungary; 7 Department of Organic and Medicinal Chemistry, Faculty of Pharmacy, University of Pécs, Honvéd Street 1, Pécs, 7624 Hungary; 8 János Szentágothai Research Center, University of Pécs, Ifjúság Street 12, Pécs, 7624 Hungary; 9 Department of Oral and Maxillofacial Surgery, University of Pécs Medical School, Tüzér Street 1, Pécs, 7623 Hungary

**Keywords:** Short fibre-reinforced composite (SFRC), Internal adaptation, Porosity, Degree of conversion, Micro-computed tomography, Micro-Raman spectroscopy

## Abstract

**Objectives:**

Evaluate and compare the internal gap (IG), closed porosity (CP), and degree of conversion (DC) of short fibre-reinforced composite (SFRC) restorations applied using various techniques in an in vitro model.

**Materials and methods:**

Five restorative protocols were tested (*n* = 20/group): (1) flowable bulk-fill SFRC; (2) flowable liner + flowable bulk-fill SFRC; (3) polyethylene fibre mesh + flowable liner + flowable bulk-fill SFRC; (4) flowable bulk-fill SFRC + high-viscosity SFRC (snowplow technique); and (5) conventional layered resin-based composite (RBC). IG (gap volume/interface volume) and CP (void volume/restoration volume) were assessed by micro-CT. DC was measured at three depths using micro-Raman spectroscopy. Data were analyzed using ANOVA, General Linear Model, and correlation tests (α = 0.05).

**Results:**

Significant differences were found among groups for IG, CP, and DC (*p* < 0.001). Groups 1, 2, and 4 showed significantly lower IG (0.46–0.53%) than Group 3 (2.78%) and Group 5 (1.09%). Group 1 demonstrated the lowest CP (0.11%), while Group 4 exhibited the highest (0.5%). DC ranged from 74.8 to 88.4%, with significant depth-related variations. Group 2 and Group 5 did not differ in average DC (*p* = 0.99).

**Conclusions:**

The flowable bulk-fill SFRC techniques demonstrated favorable internal adaptation, low CP, and acceptable DC, supporting their potential use in deep cavities. In contrast, polyethylene fibre mesh reinforcement increased IG formation, whereas the snowplow technique achieved high DC at the expense of increased CP.

**Clinical Relevance:**

Flowable bulk-fill SFRC may improve internal adaptation, reduce porosity, and achieve acceptable DC in deep cavities. Polyethylene fibre mesh and snowplow techniques exhibit technique-dependent limitations.

## Introduction

Dental resin-based composites (RBCs) are widely used as direct esthetic restorative materials for the rehabilitation of extensive posterior lesions [1]. However, despite significant advances in material formulations and curing protocols, polymerization shrinkage remains a fundamental limitation that compromises interfacial integrity, an essential determinant of long-term clinical performance [2, 3]. The stresses generated during polymerization may result in gap formation, microcracks, postoperative sensitivity, marginal discoloration, and recurrent caries [4, 5]. Among these complications, interfacial gaps and internal voids are particularly relevant because they impair the mechanical stability of the restoration and may facilitate fluid movement through dentinal tubules, thereby contributing to postoperative sensitivity [6–8].

The degree of conversion (DC) is a critical parameter affecting the mechanical properties, wear resistance, and biocompatibility of RBCs. However, higher DC values are also associated with increased polymerization shrinkage and stress development, which may adversely affect internal adaptation [9]. In addition to polymerization kinetics, gap formation is influenced by cavity configuration, material composition, restorative technique, and light-curing conditions [10].

Deep Class II mesio-occlusal-distal (MOD) cavities represent a particularly challenging clinical situation. Their large volume and complex geometry increase the risk of inadequate adaptation and void formation, while the loss of supporting tooth structure may further compromise long-term restoration performance [11–17].

Various strategies have been proposed to reduce polymerization stress and improve adaptation, including modifications in resin chemistry, stress modulators, optimized curing protocols, incremental placement, bulk-fill techniques, and the use of flowable liners [3, 18–23]. More recently, short fibre-reinforced composites (SFRCs) have attracted considerable interest because of their favorable mechanical properties and reduced polymerization shrinkage compared with conventional RBCs [24, 25]. Another proposed approach is reinforcement with ultra-high molecular weight polyethylene fibres (Ribbond-Ultra), which may alter stress distribution within the restoration and redirect forces away from the bonded interface [26, 27].

Despite these developments, limited information is available regarding how different SFRC application techniques influence internal adaptation, porosity, and degree of conversion in deep MOD restorations. Therefore, the present in vitro study evaluated and compared five restorative approaches: (1) bulk-filled flowable SFRC, (2) flowable liner combined with bulk-filled flowable SFRC, (3) polyethylene fibre mesh incorporated into a flowable liner beneath bulk-filled SFRC, (4) the snowplow technique combining flowable and packable SFRC, and (5) conventional incremental layering with a conventional RBC.

The objective of this in vitro study was to evaluate and compare the internal gap volume (IG), closed porosity (CP), and degree of conversion (DC) of these restorative techniques using micro-computed tomography and micro-Raman spectroscopy. The following null hypotheses were tested: (1) restorative technique would not influence IG or CP; (2) DC would not differ among restorative techniques or restoration depths; and (3) no significant correlations would exist among IG, CP, DC.

## Materials and methods

### Specimen preparation

The present study received ethical approval from both the Ethics Committee of the University of Szeged and the Hungarian Medical Research Council (Approval number: BM/23566-1/2023) and was conducted in full accordance with the principles of the Declaration of Helsinki. Mandibular third molars that had been extracted for orthodontic reasons were selected for inclusion in the study. The teeth exhibited uniform coronal dimensions: the orovestibular width ranged between 9 and 10 mm, the mesiodistal width between 10 and 11 mm, and the crown height—measured from the cemento-enamel junction (CEJ)—was between 6 and 8 mm. Throughout the study period, all specimens were stored in 0.1% thymol solution at 4 °C. The teeth were used within six months of extraction.

Standardized MOD cavities were prepared in all teeth selected for the study. The preparation protocol followed the method established in a previous study [5, 28]. The cavities were shaped to a uniform depth of 5 mm, with both buccal and lingual wall thicknesses set at 2.5 mm. The experimental procedure involved the utilisation of a round-end parallel diamond bur (881.31.014 FG – Brasseler USA Dental, Savannah, GA, USA). This instrument was initially positioned at the centre of the occlusal surface, a point that was determined by bisecting the distance between the buccal and lingual cusp tips. During the process of cavity preparation, a digital caliper manufactured by Mitutoyo Corp. (Kawasaki, Japan) was utilised to undertake continuous monitoring of the wall thickness at the base of the cavity, thereby ensuring the maintenance of the targeted thickness of 2.5 mm. The preparation of the walls was undertaken in a manner parallel to the long axis of the tooth. The measurement of cavity depth was conducted from the cusp tip to the base, utilising a 15 UNC periodontal probe (Hu-Friedy Mfg. Co., Chicago, USA). This ensured that the probe maintained firm contact with the surface of the tooth during the measurement process. Each final cavity was constituted of a single, continuous unit (as a slot), with the proximal box corresponding to the occlusal portion in both width and depth. All cavity walls were prepared without bevels, aiming to produce butt-joint cavosurface margins.

Following the preparation of the cavities, each tooth was subjected to a thorough examination under 4.3x magnification using a D-Light Pro device (GC Europe, Leuven, Belgium) in detection mode to identify the presence of any enamel fractures. Specimens exhibiting pre-existing cracks were excluded from the study and substituted with intact third molars, which subsequently underwent the same cavity preparation process. In the final analysis, 100 MOD-prepared mandibular third molars were included in the study and randomly assigned to five experimental groups (*n* = 20 per group).

### Restorative procedures

All teeth underwent a standardized adhesive procedure. A Tofflemire matrix band (1101 C 0.035, KerrHawe, Bioggio, Switzerland) was placed, after which the enamel margins were selectively etched with 37% phosphoric acid for 15 s, followed by thorough rinsing with water. The cavity was gently dried to remove water pooling after rinsing, and a one-step self-etch adhesive (G-Premio Bond, GC Europe, Leuven, Belgium) was applied in accordance with the manufacturer’s guidelines. G-Premio Bond was selected because its compatibility with the restorative materials investigated allowed standardization of the adhesive procedure across all experimental groups. Given the deep MOD cavity design, the adhesive polymerization was performed using an extended 60-second exposure time with a quartz-tungsten-halogen light-curing unit (Optilux 501, Kerr Corp., Orange, CA, USA; glass light guide with an exit window diameter: 11 mm, wavelength range: 400–505 nm) in continouos mode to minimize the risk of insufficient adhesive polymerization and to standardize the bonding procedure across all experimental groups. The device’s average irradiance, as verified with a digital radiometer (Bluephase Meter II, Ivoclar Vivadent, Schaan, Liechtenstein), was 820 ± 40 mW/cm². To ensure consistent light-curing performance, the irradiance was re-measured after every fifth restoration using the same radiometer.

Following the standardized adhesive protocol, the restorative procedures used in the five experimental groups were as follows:

- **Group 1**. The cavity was restored using a bulk increment of flowable SFRC (bulk shade) as the core build-up. A 1-mm-thick coronal covering layer of flowable SFRC in dentin shade was then applied to complete the restoration. Each layer was light-cured for 20 s using the quartz-tungsten-halogen curing unit described above.
- **Group 2.** A flowable conventional RBC (U shade) was first placed as a 0.5–1 mm thin liner at the cavity base. The cavity was then restored with a bulk increment of flowable SFRC (bulk shade) as the core material, and a 1-mm-thick coronal covering layer of flowable SFRC in dentin shade was applied. Each layer was light-cured for 20 s using the quartz-tungsten-halogen curing unit described above.
- **Group 3.** Polyethylene fibres were embedded in a flowable conventional RBC base placed at the bottom of the cavity in a 0.5–1 mm layer thickness. Thereafter, the cavity was restored with a bulk increment of flowable SFRC (bulk shade) as the core material, and the restoration was completed with a 1-mm-thick coronal covering layer of flowable SFRC in dentin shade. Each layer was light-cured for 20 s using the quartz-tungsten-halogen curing unit described above.
- **Group 4.** The restoration was performed using the snowplow technique, in which flowable SFRC (bulk shade) and bulk-fill packable SFRC (bulk shade) were placed in combination and light-cured for 20 s, followed by the application and light-curing of the 1-mm-thick coronal covering layer of flowable SFRC in dentin shade for 20 s using the quartz-tungsten-halogen curing unit described above.
- **Group 5.** The cavity was restored with conventional packable RBC (A2 shade) using an oblique incremental layering technique. The material was applied in approximately 2-mm-thick oblique increments until the cavity was completely restored. Each increment was light-cured for 20 s using the curing unit described above.

For improved readability, the five restorative protocols are hereafter referred to as Flow SFRC (Group 1), Liner + Flow SFRC (Group 2), Fibre mesh + Flow SFRC (Group 3), Snowplow SFRC (Group 4), and Layered RBC (Group 5).

**Fig. 1:**
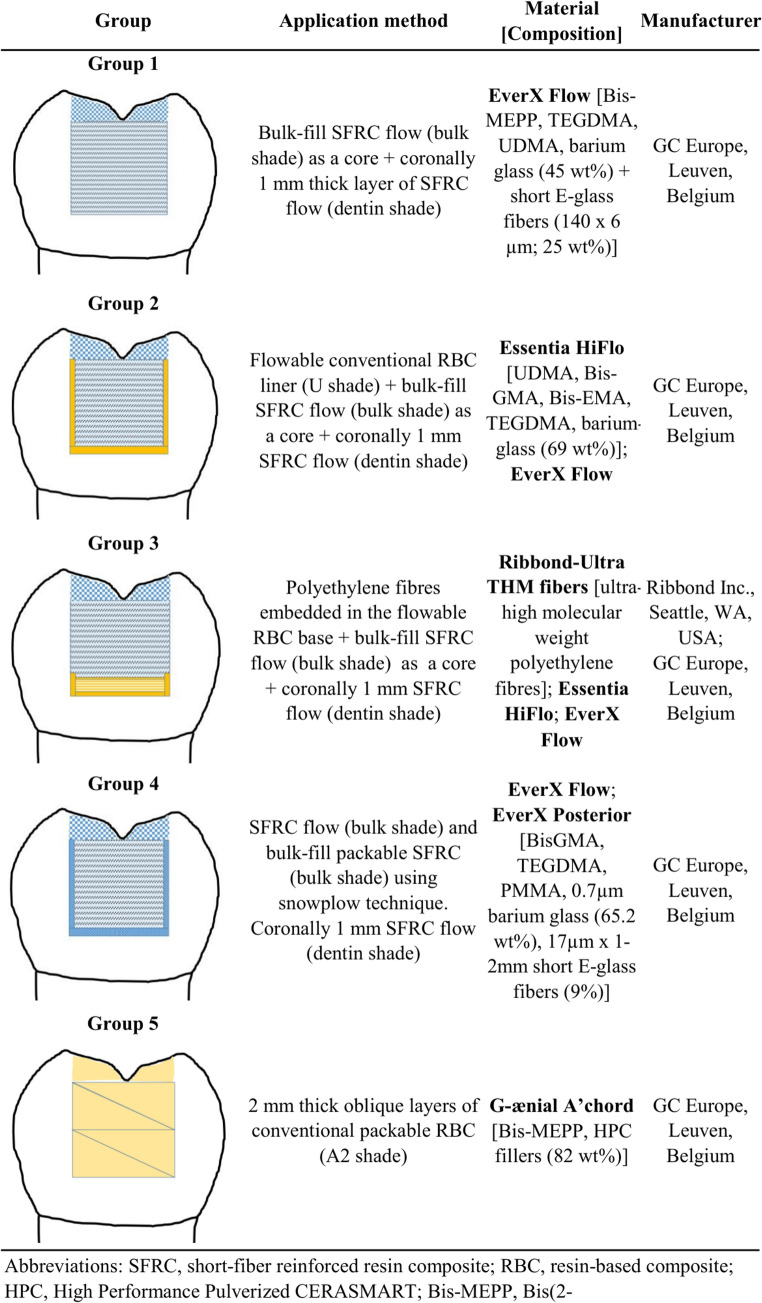
Study groups, application methods, materials and manufacturers

Figure 1 shows the application methods, and enumerates the materials employed, their composition, and the respective manufacturers.

### Micro-computed tomography measurement − 3D internal adaptation and porosity

The IG and CP of restorations fabricated using various application techniques of SFRC were evaluated using micro-CT following one month of wet storage. Each sample (*n* = 5 × 20) was centrally aligned in the field of view, with the scanning axis positioned parallel to the tooth’s axial plane. Micro-CT imaging was performed using a Skyscan 1176 system (Bruker, Kontich, Belgium), operated with software version 1.1 (build 12). The scans were acquired at a tube voltage of 80 kV and a current of 310 µA, with an exposure time of 1500 ms. The reconstructed isotropic voxel size was 8.74 μm. A 1-mm aluminum filter was used during image acquisition, and the total scan duration was approximately 35 min per sample. Image reconstruction and processing were conducted with a dedicated software suite. NRecon (version 1.7.4.2) was used for primary image reconstruction, while DataViewer (version 1.5.6.2, 64-bit) facilitated multiplanar reformatting and orientation adjustment. Subsequent image analysis and quantitative processing were carried out in CTan (version 1.20.8.0+), and final 3D visualization and volume rendering were performed using CTvox (version 3.1.1 r1191, 64-bit). Reconstruction parameters included a ring-artifact correction level of 20, no edge smoothing (value = 0), and a radius gain of 20%.

For IG evaluation, the region of interest (ROI) was defined as a 0.1-mm tooth region combined with a 0.1-mm restoration region at the tooth–restoration interface. For the CP assessments, the entire restoration served as the ROI. Prior to segmentation, Gaussian low-pass filtering was applied for noise reduction. Thresholding was performed using a global threshold method to generate binary images. Binarization converted the grayscale datasets into black-and-white images to differentiate object structures from the background. Gap and void identification was based on thresholding for air-equivalent density regions. Internal gap quantification was expressed as the ratio of the interfacial gap volume to the total ROI volume (IG%). Closed porosity quantification was defined as the internal void volume relative to the total restoration volume (CP%).

### Micro-Raman spectroscopy measurements – degree of conversion

The DC was assessed after one month of wet post-curing storage using a confocal Raman spectrometer (Labram HR 800, HORIBA Jobin Yvon S.A.S., Longjumeau Cedex, France). Five teeth from each group were sectioned in a vertical plane parallel to its mesio-distal axis using a water-cooled diamond blade (Isomet Diamond Wafering Blade, no. 11–4244, Buehler Ltd., Lake Buff, IL, USA). Following a polishing procedure (Sof-Lex Polishing Disc Kit, 3 μm, St. Paul, MN, USA) and a cleaning process (ultrasonic bath for 10 min; Emmi-20HC, eMAG, Salach, Germany), the sectioned specimens were affixed to a universal sample holder that allowed controlled translation along the z-axis. This facilitated the acquisition of Raman spectra at multiple depths, including 0.5 mm below the surface (top); at the geometric centre of the distance between the top and bottom of the sample (middle); and 0.5 mm occlusally from the bottom of the cavity (bottom). For each region, three points were analysed, and ten scans per point were collected, with the averaged spectra used for evaluation. The exposed measurement area had a diameter of approximately 0.2 mm, and each scan was acquired with an integration time of 10 s. A 20 mW He–Ne laser operating at 632.817 nm served as the excitation source. Spectra were obtained using a ×100 objective lens (Olympus UK Ltd.), yielding a spatial resolution of roughly 15 μm and a spectral resolution of about 2.5 cm⁻¹. Diffraction gratings of 600 l/mm and 1800 l/mm were employed. Signal detection was achieved using a Peltier-cooled CCD detector with a resolution of 1024 × 256 pixels.

Unpolymerized RBC specimens were also measured to serve as reference spectra. Spectral processing—including baseline correction, background subtraction, and range selection (1440–1660 cm⁻¹)—was conducted using LabSpec 5.0 (HORIBA). An eighth-order polynomial was fitted to the baseline and subtracted from the raw data. Lorentzian peak fitting was applied at 1458, 1609, and 1640 cm⁻¹ using Origin software (Microcal Software Inc., Northampton, MA, USA) to determine peak intensities.

The DC% was calculated by assessing the reduction in the intensity of the aliphatic C = C peak (1640 cm⁻¹) relative to a reference peak before and after polymerization. For EverX Posterior and Essentia HiFlo the aromatic C = C peak at 1609 cm⁻¹ served as the reference. In contrast, for EverX Flow and G-aenial A’chord, which lack aromatic structures, the CH₂ deformation band at 1458 cm⁻¹ was used. The DC was computed using the following formula:

$$DC\%=(1-R_{cured}/R_{uncured})\times100$$

where the term R is employed to denote the ratio of peak intensities at 1640 cm^− 1^ and 1609 cm^− 1^ or 1458 cm^− 1^ (as references) associated with unconjugated and conjugated carbon bonds or CH_2_ deformation in non-polymerised and polymerised RBCs, respectively.

### Statistical analysis

The sample size was determined based on a previously published study [28], which employed a similar experimental design and methodology to evaluate IG and DC.

Sample size formula:

   

$$\:n=\frac{{(z_{1-\frac{\alpha\:}2}+z_{1-\beta\:})}^2{(s_1+s_2)}^2}{(M_1-{M_2)}^2}$$

[z = standard score; α = probability of Type I error at 95% confidence level = 0.05; z_1−α/2_ = 1.96 for 95% confidence; β = probability of Type II error = 0.20; 1 − β = the power of the test = 0.80; z_1−β_ = value of standard normal variate corresponding to 0.80 value of power = 0.84; s_1_ = standard deviation of the outcome variable of group 1; s_2_ = standard deviation of the outcome variable of group 2; M_1_ = mean of the outcome variable of group 1; M_2_ = mean of the outcome variable of group 2. For IG determination the s_1_ = 0.03; s_2_ = 0.04; M_1_ = 0.47; M_2_ = 0.42. For DC determination the s_1_ = 1.4; s_2_ = 2.6; M_1_ = 83.2; M_2_ = 77.0. In accordance with the findings of the calculations, the sample size was determined to be *n* = 20 and *n* = 5 for IG/CP and DC measurements, respectively, within each group.

The normality of the data was assessed using the Shapiro–Wilk test, and homogeneity of variances was evaluated using Levene’s test. For repeated measures ANOVA, the assumption of sphericity was assessed, and when violated, the Greenhouse–Geisser correction was applied. Parametric analyses were used when the relevant assumptions were considered sufficiently met. In cases of minor deviation from these assumptions, the data were carefully inspected and the interpretation of the findings was supported by complementary analyses, as described below.

A one-way analysis of variance (ANOVA) was employed to analyse the differences in IG, CP, and average DC among the five restorative techniques. This was followed by Tukey’s post hoc test for pairwise comparisons. In order to evaluate intra-group differences in DC across the top, middle, and bottom regions of the restorations, a repeated measures ANOVA was conducted. Subsequent post hoc comparisons were conducted using Tukey’s test to adjust for multiple comparisons and control the familywise error rate. Data were also analyzed using General Linear Model (GLM) to evaluate the effect of restorative technique on each dependent variable (IG, CP, and DC). In addition, linear regression analyses were performed to assess the strength and proportion of variance explained (R², adjusted R²) between the restorative approach and the measured outcomes. Effect sizes were reported as partial η² for GLM and R² for regression analyses. Finally, associations among the studied parameters were evaluated in the pooled dataset using Spearman’s rank correlation coefficient. Although several variables showed approximately normal distributions within the individual restorative groups, the pooled data used for the correlation analyses deviated significantly from normality according to the Shapiro–Wilk test. Therefore, a non-parametric correlation approach was considered more appropriate. The statistical significance was set at *p* < 0.05 for all analyses.

## Results

### Micro-computed tomography measurement − 3D internal adaptation and porosity

**Fig. 2:**
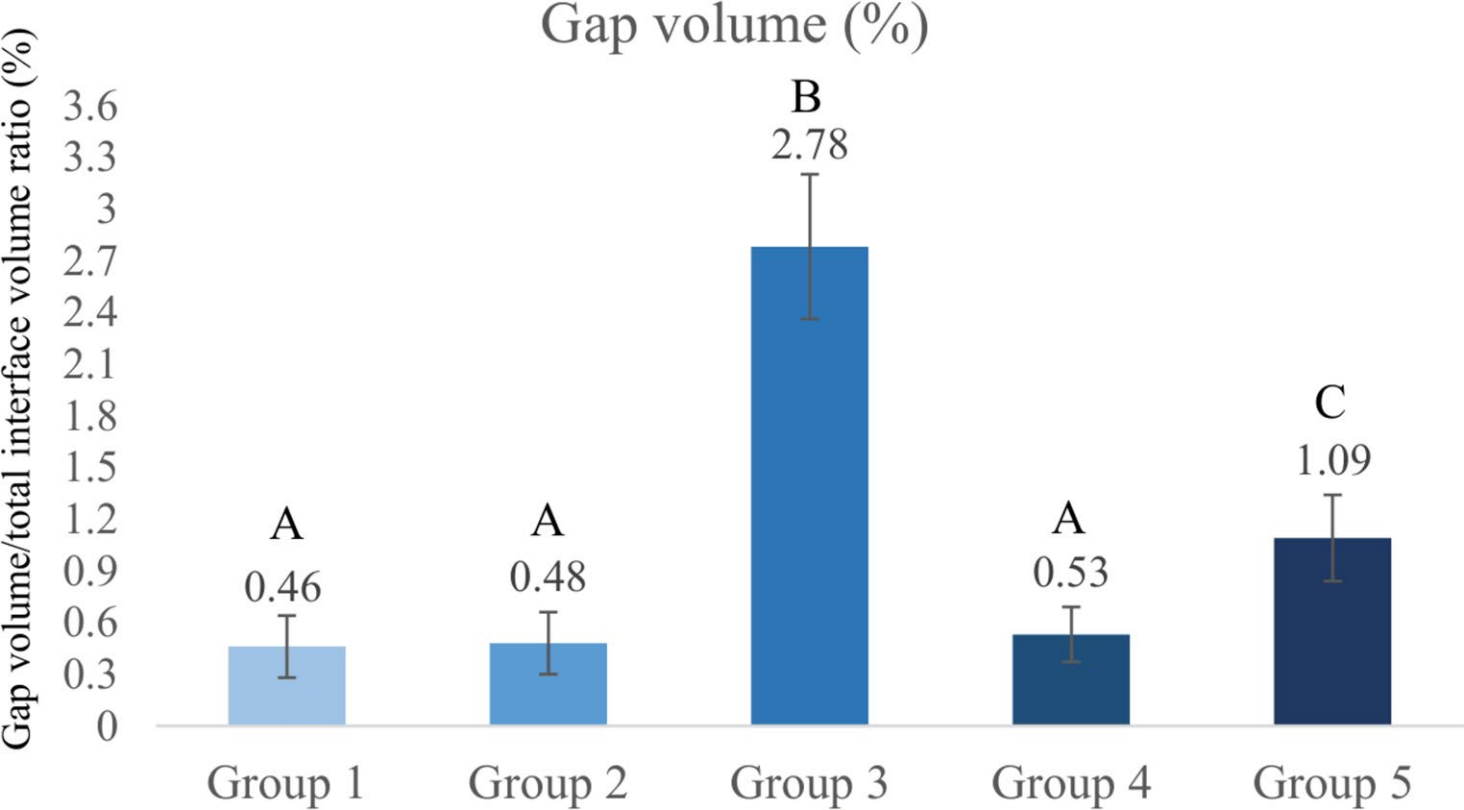
The ratio of the interfacial gap volume to the total volume of the examined region of interest (ROI) was evaluated using 3D micro-computed tomography measurements. Different capital letters indicate statistically significant differences according to one-way ANOVA and Tukey’s post hoc tests

**Fig. 3:**
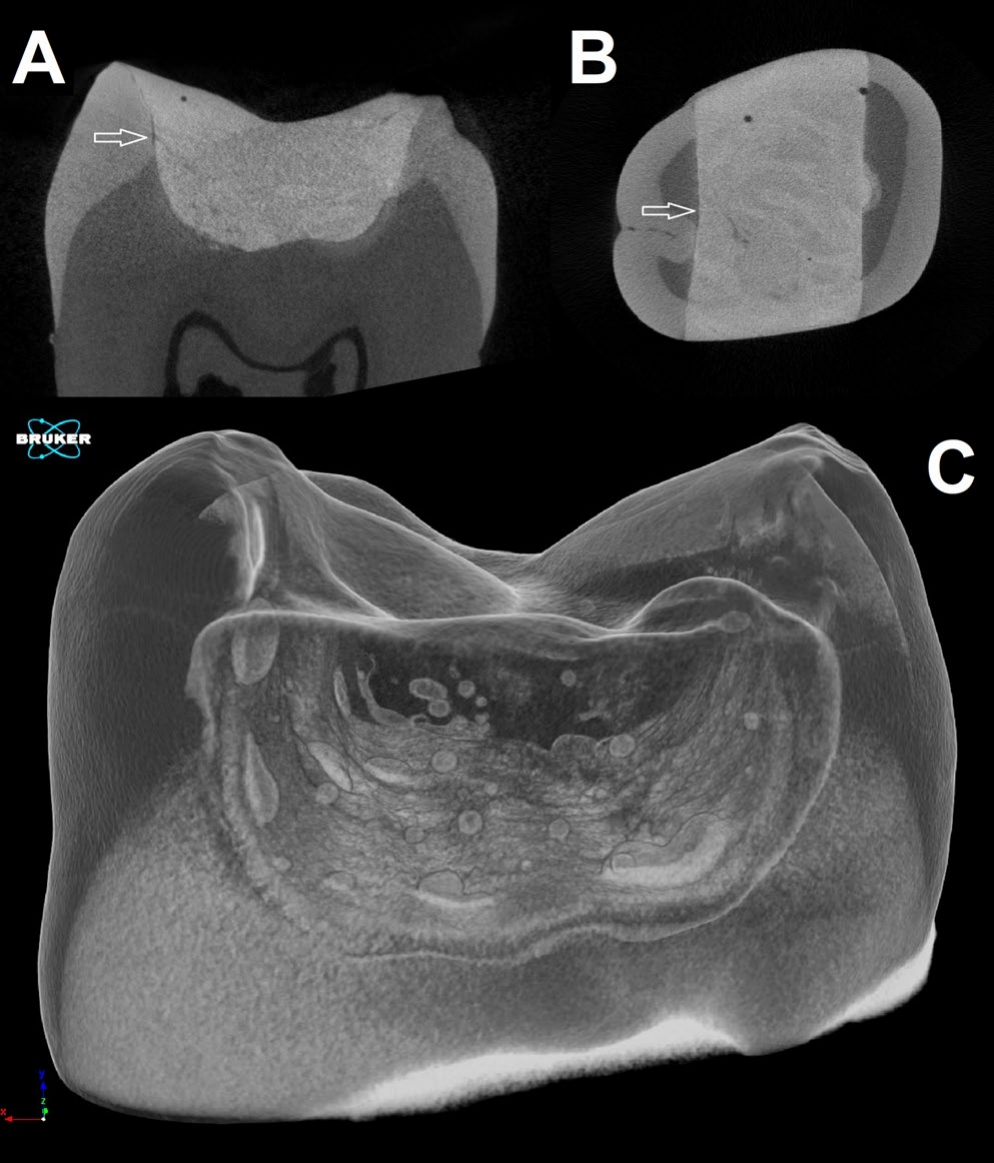
Representative micro-CT images of Group 1 (Flow SFRC). Figure **A** shows a coronal slice, while figure **B** shows an axial slice of the 2D image of the restored tooth. Figure **C** presents a 3D coronal image. Arrows indicate the formation of internal gaps along the tooth-restoration interface

**Fig. 4:**
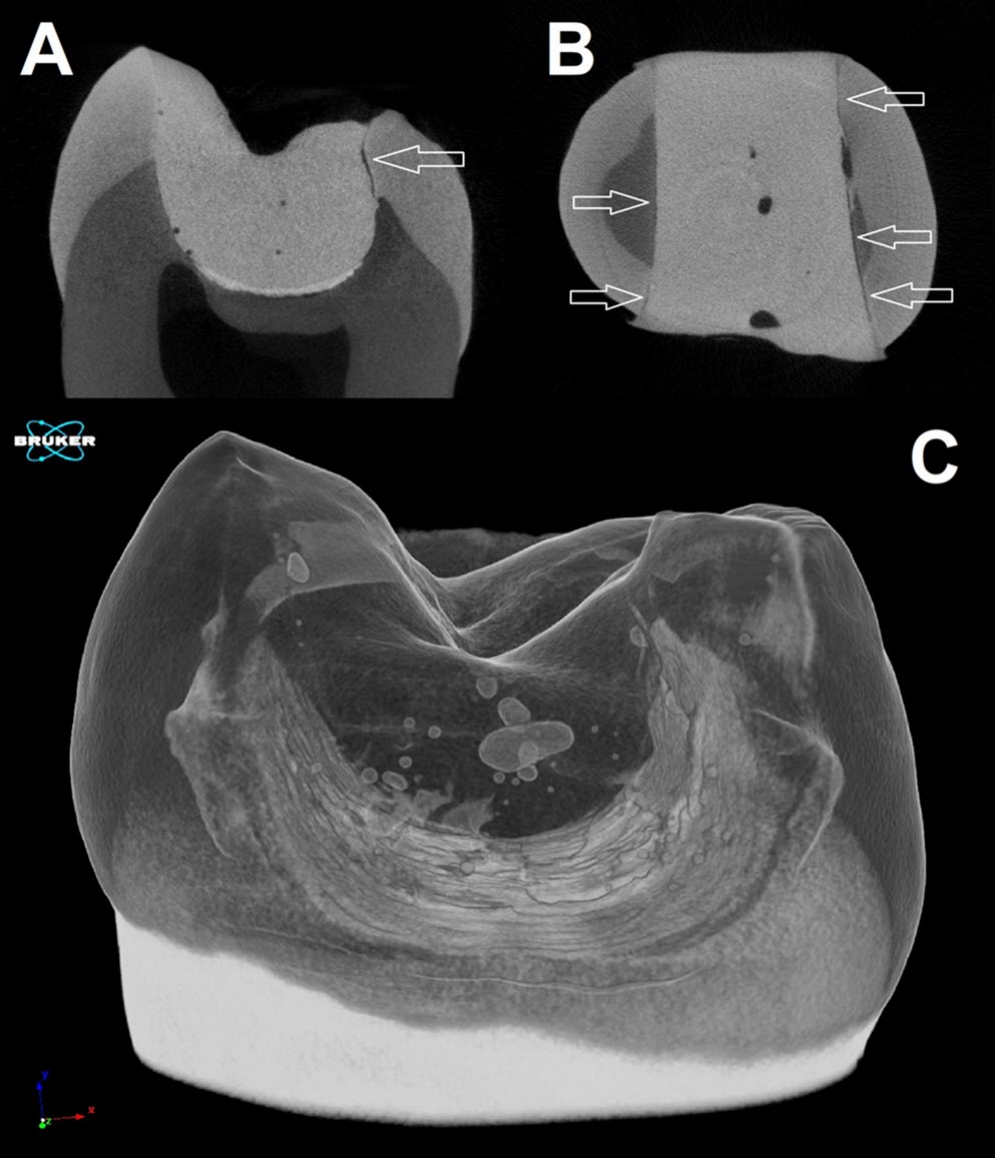
Representative micro-CT images of Group 2 (Liner + Flow SFRC). Figure **A** shows a coronal slice, while figure **B** shows an axial slice of the 2D image of the restored tooth. Figure **C** presents a 3D coronal image. Arrows indicate the formation of internal gaps along the tooth-restoration interface

**Fig. 5:**
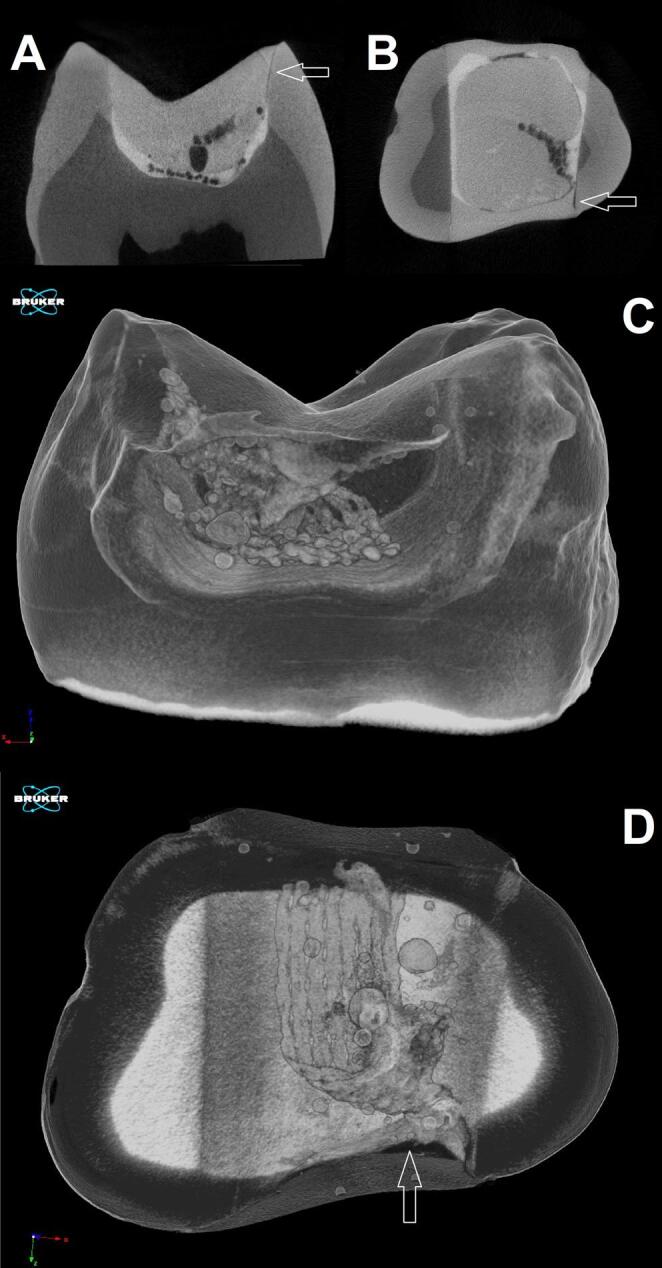
Representative micro-CT images of Group 3 (Fibre mesh + Flow SFRC). Figure **A** shows a coronal slice, while figure **B** shows an axial slice of the 2D image of the restored tooth. Figure **C** presents a 3D coronal image, while figure **D** shows an axial image of a tooth from Group 3, in which the restoration was executed using Essentia HiFlo as a liner embedding Ribbond polyethylene fibre mesh, EverX Flow with bulk application, overlaid with 1 mm EverX Flow dentin shade. Note that one end of the Ribbon mesh folds back, creating numerous voids in EverX Flow. Arrows indicate the formation of internal gaps along the tooth-restoration interface

**Fig. 6:**
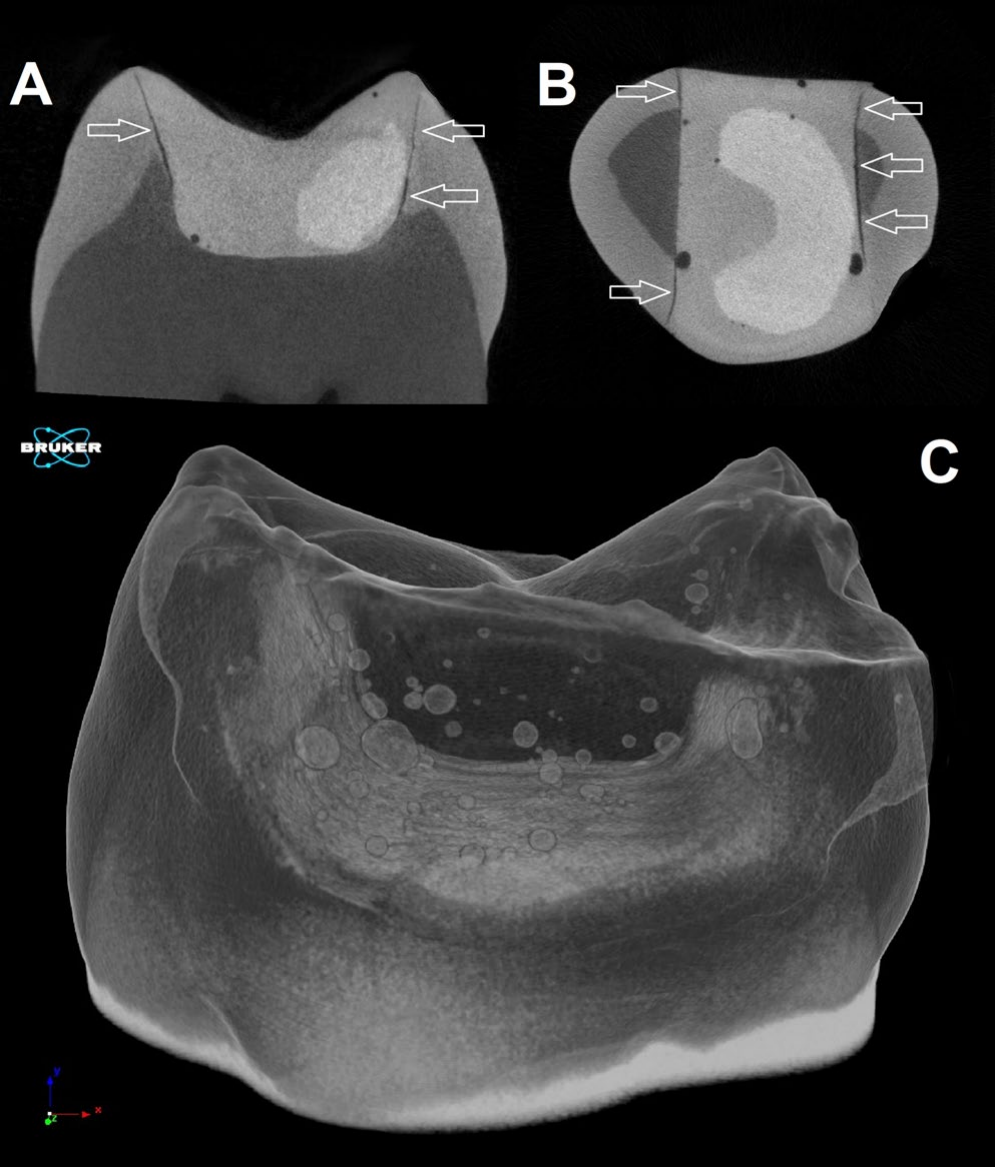
Representative micro-CT images of Group 4 (Snowplow SFRC). Figure **A** shows a coronal slice, while figure **B** shows an axial slice of the 2D image of the restored tooth. Figure **C** presents a 3D coronal image. Arrows indicate the formation of internal gaps along the tooth-restoration interface

**Fig. 7:**
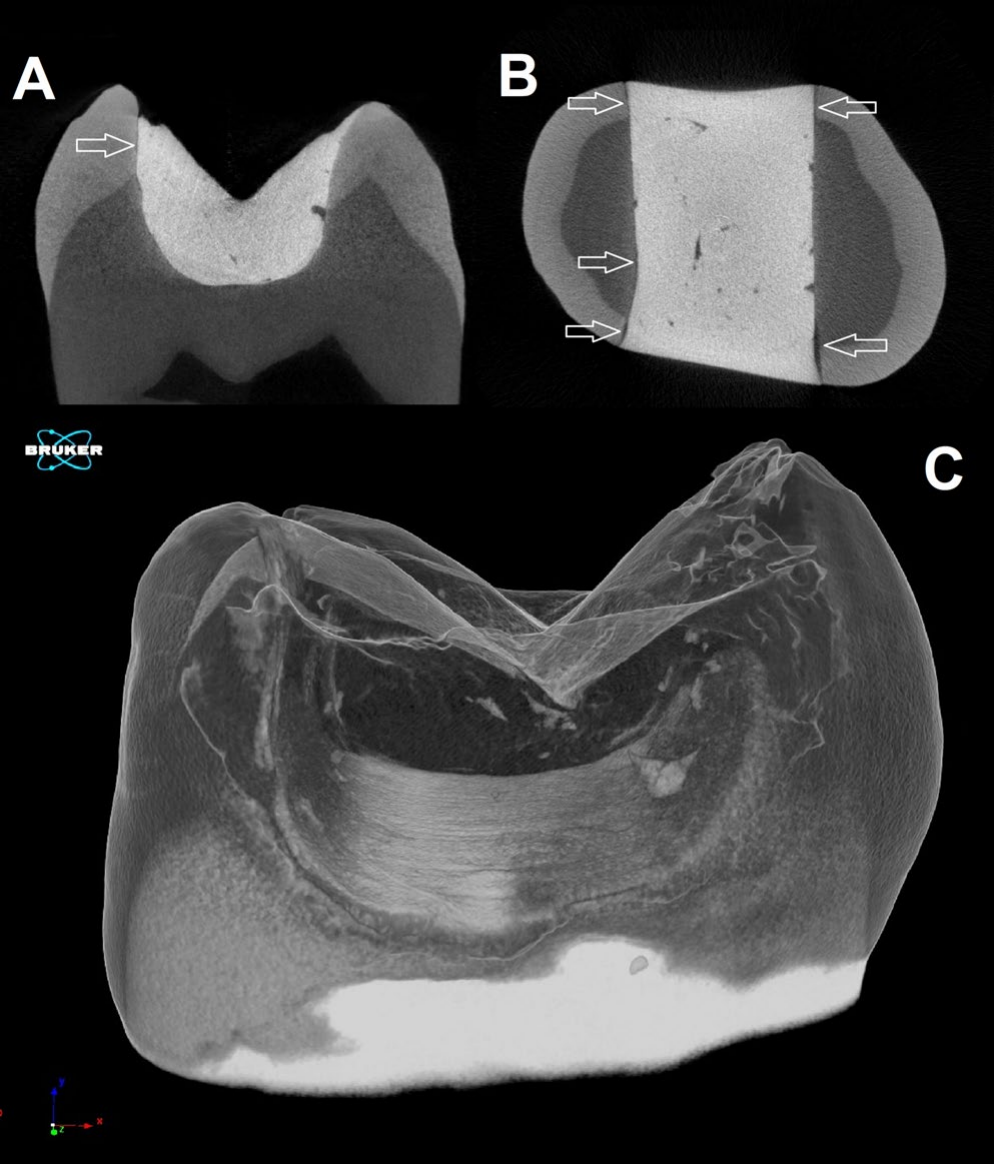
Representative micro-CT images of Group 5 (Layered RBC). Figure **A** shows a coronal slice, while figure **B** shows an axial slice of the 2D image of the restored tooth. Figure **C** presents a 3D coronal image. Arrows indicate the formation of internal gaps along the tooth-restoration interface

The ratio of interfacial gap volume (IG%) to the total interface volume is shown in Fig. 2. One-way ANOVA revealed significant differences among the five restorative groups (*p* < 0.001). Group 3 (Fibre mesh + Flow SFRC) exhibited the highest IG values, whereas Groups 1, 2, and 4 showed the lowest and did not differ significantly from each other. Group 5 (Layered RBC) showed intermediate IG values, significantly higher than those of Groups 1, 2, and 4, but lower than those of Group 3 (Fibre mesh + Flow SFRC) (Figs. 3, 4, 5, 6 and 7).

GLM analysis confirmed a significant effect of restorative technique on IG (F(4,80) = 131.48, *p* < 0.001, partial η² = 0.87), indicating a strong influence of application method on internal adaptation.

**Fig. 8:**
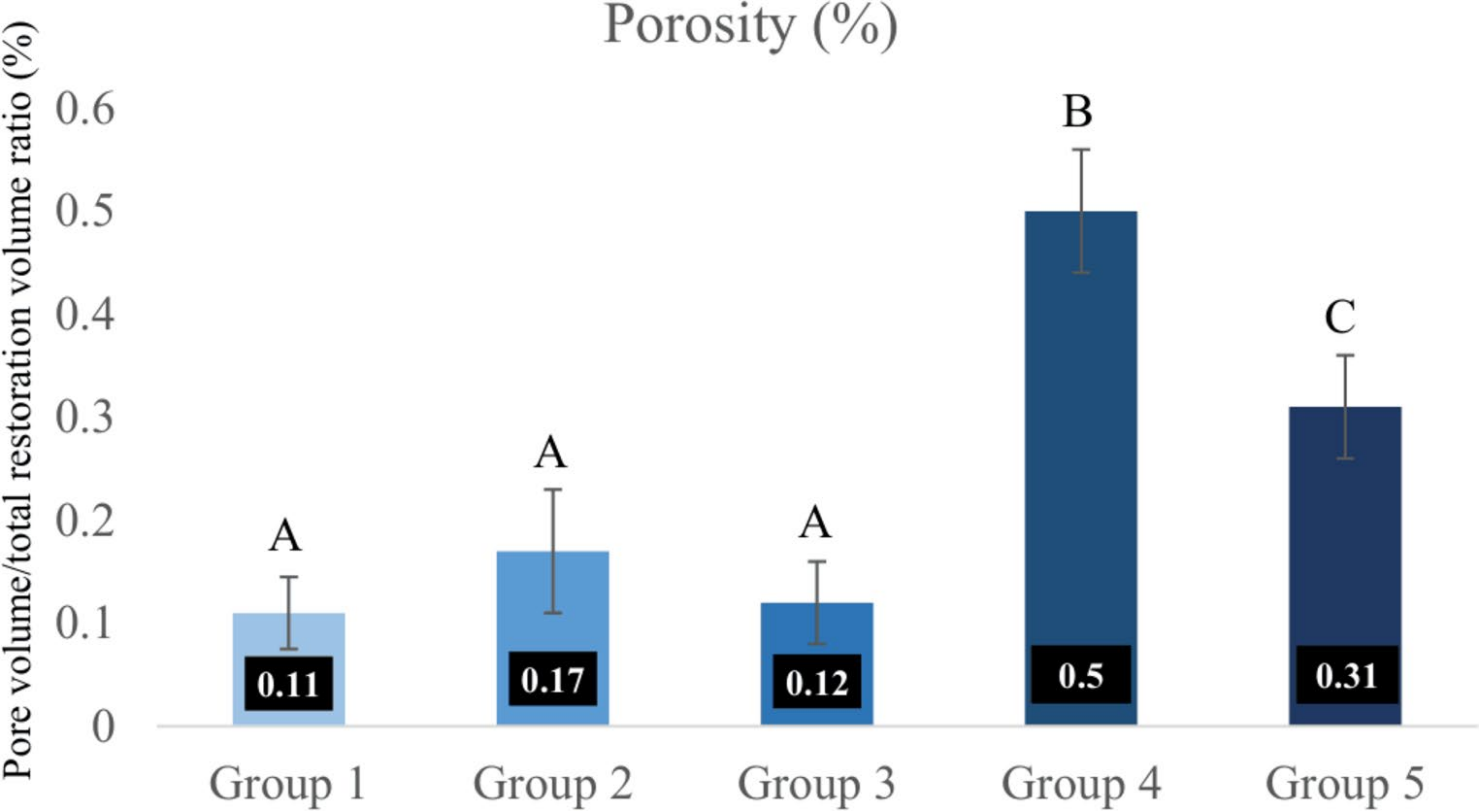
The ratio of the closed pore volume to the total volume of the restoration as region of interest (ROI) was evaluated using 3D micro-computed tomography measurements. Different capital letters indicate statistically significant differences, as determined by one-way ANOVA and Tukey’s post hoc tests

The closed porosity (CP%) values are presented in Fig. 8. One-way ANOVA demonstrated significant differences among the groups (*p* < 0.001). Group 4 (Snowplow SFRC) showed the highest porosity values. Groups 1, 2, and 3 exhibited similarly low CP values and did not differ significantly from one another. Group 5 (Layered RBC) showed intermediate CP values, being significantly higher than Groups 1 and 3, but lower than Group 4 (Snowplow SFRC).

GLM analysis also showed a significant effect of restorative technique on CP (F(4,80) = 28.79, *p* < 0.001, partial η² = 0.59). No significant correlation was found between IG and CP (Spearman’s ρ = 0.25, *p* = 0.19).

### Micro-Raman spectroscopy measurement – degree of conversion

**Fig. 9:**
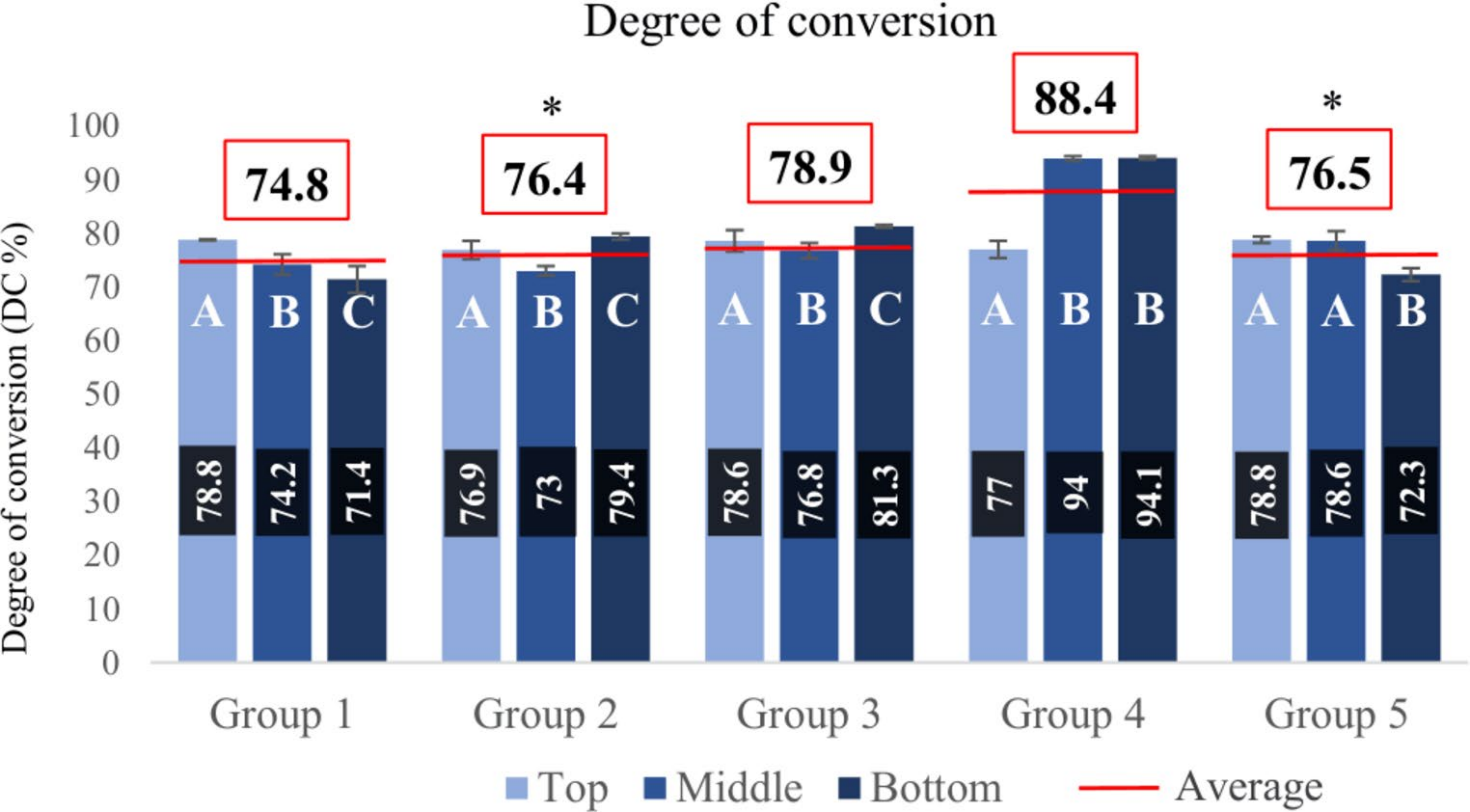
The degree of conversion was measured at the top, middle and bottom of the halved resin composite restorations. Different capital letters indicate a statistically significant difference within one group, as determined by repeated measures ANOVA. An asterisk (*) indicates that the average degree of conversion for Group 2 (Liner + Flow SFRC) and 5 (Layered RBC) did not differ significantly (repeated measures ANOVA and Tukey’s post hoc test)

The DC% values are shown in Fig. 9. Repeated measures ANOVA with Greenhouse–Geisser correction revealed a significant effect of depth on DC in all groups (all *p* ≤ 0.001), indicating that polymerization varied significantly across the top, middle, and bottom regions of the restorations.

Post hoc comparisons showed that in Group 1 (Flow SFRC), DC decreased significantly with increasing depth (top > middle > bottom; all *p* < 0.001). In Groups 2 and 3, the top and middle values did not differ significantly, whereas the bottom layer showed significantly higher DC than the other regions. In Group 4 (Snowplow SFRC), the middle and bottom regions did not differ significantly, but both exhibited significantly higher DC than the top region. In Group 5 (Layered RBC), the bottom layer showed significantly lower DC than the top and middle layers, while the latter two did not differ significantly.

One-way ANOVA demonstrated a significant effect of restorative technique on average DC (*p* < 0.001). Post hoc analysis showed significant differences between all groups except Groups 2 and 5, which did not differ significantly. GLM analysis confirmed a significant effect of restorative technique on average DC (F(4,40) = 741.8, *p* < 0.001, partial η² = 0.99).

Spearman’s rank correlation analysis revealed a significant moderate positive correlation between IG and DC (ρ = 0.31, *p* = 0.04), as well as between CP and DC (ρ = 0.34, *p* = 0.02).

## Discussion

The findings of this in vitro study indicate that the type of material and application protocol significantly influences IG, CP, and DC profiles. These results provide critical insights for selecting restorative techniques in structurally compromised posterior teeth.

The first null hypothesis, which posited no significant differences in IG among the tested techniques, was rejected. The generation of three-dimensional models was facilitated by the utilisation of micro-CT scan data, thereby enabling precise volume calculations. Micro-CT is widely utilised for its accuracy and reliability in the field of dentistry, particularly in the analysis of the tooth-restoration interface and the quantification of gaps and voids. Among the tested application methods, Groups 1, 2, and 4 achieved the most favorable IG in all of which utilized SFRC in bulk-fill applications—either alone or in combination with liners or in the snowplow technique. These groups demonstrated significantly lower IG volumes compared to Group 3 (Fibre mesh + Flow SFRC) and Group 5 (Layered RBC). It was demonstrated, that a fibre-reinforced increment acts as a shrinkage stress breaker and protects the bonded interface at deep dentin [29]. Long polyethylene fibres have been shown to be especially effective in absorbing and redistributing masticatory forces in teeth with significant dentin loss [30]. Consequently, it is anticipated that these fibres will exhibit enhanced fracture resistance in large MOD cavities. However, in our study, Group 3 (Fibre mesh + Flow SFRC) exhibited the highest interfacial gap formation, which exceeded fivefold that of Groups 1, 2, and 4. The findings may be attributed to the inclusion of the Ribbond-Ultra fibre mesh, which could have interfered with the flow and adaptation of the SFRC, resulting in voids and incomplete interface contact. As was evidenced in earlier studies, the incorporation of fibres without adequate wetting or adaptation has been shown to result in a deterioration of interface quality [29, 31]. Whilst there is considerable potential for polyethylene fibre reinforcement to offer significant benefits, its clinical adoption may be limited by complex application protocols and restoration designs. According to recent studies and randomised clinical trials, such designs often fail to significantly improve outcomes, whilst concomitantly introducing increased handling difficulty, cost, and chair time [32].

In contrast, the snowplow technique employed in Group 4 (Snowplow SFRC) yielded commendable IG outcomes. This restorative method involves the concurrent placement of a layer of uncured flowable RBC, followed immediately by a more viscous RBC. The two layers are then light-cured simultaneously [33]. The hypothesis is that this technique may enhance the adaptation process by allowing the low-viscosity flowable SFRC to better infiltrate cavity irregularities before being displaced and compacted by the more viscous packable SFRC [34]. This method aligns with previous findings emphasizing the benefits of dynamic layering techniques for improved marginal and internal seal [35].

The IG observed within the control group (Group 5 - Layered RBC), was found to be substantially less effective, exhibiting values twice the level exhibited by groups 1, 2, and 4. The extant literature does not present a consistent picture regarding the effect of bulk or layered filling techniques on internal adaptation. The findings of various studies suggest that layering is more advantageous [36], while others favour the bulk technique [28] or indicate similar performance when the two techniques are compared [37]. However, an additional distinguishing feature of the bulk-fill RBCs employed in our study is their incorporation of short glass fibres. SFRCs can decrease polymerization stress – a factor potentially responsible for IG formation - due to the inclusion of short glass fibres, which can absorb and dissipate the stress generated during the polymerization process, leading to comparable or reduced marginal gaps and improved adaptation compared to conventional RBCs.

The second aspect of the first null hypothesis, regarding CP, was also rejected. Significant differences in CP were detected across groups. The strong and statistically significant association between the restorative technique and CP suggests that the material layering strategy and handling method substantially influence the formation of internal voids within the restoration. With an adjusted coefficient of determination of 0.32, the restorative configuration explained about 32% of the variance in porosity, indicating that application technique plays a key role in the structural integrity of SFRC restorations. Nevertheless, other factors such as material viscosity, fibre distribution, and operator handling may also contribute to the observed variability. Despite demonstrating excellent adaptation and DC, Group 4 (Snowplow SFRC) exhibited the highest porosity levels among all the tested materials. The placement of a highly viscous and sticky SFRC material could potentially trap air bubbles during insertion, leading to closed voids. As demonstrated in previous micro-CT studies, the employment of high viscosity RBCs in bulk-pack techniques is associated with an increased internal porosity. This is due to the limitation of venting pathways during material condensation [38]. In contrast, Groups 1, 2, and 3 exhibited low and comparable porosity values, suggesting that the use of a low viscosity SFRC material in a bulk-fill application may better facilitate homogenous material distribution. Although Group 3 (Fibre mesh + Flow SFRC) showed high IG volumes, the porosity within the bulk restoration remained low—possibly due to the high flowability of the liner and the bulk RBC. However, as demonstrated in Fig. 5, the mesh can exhibit consolidation, resulting in the formation of air pockets surrounding the mesh within the flowable RBC [29]. It is evident that adapting this necessitates meticulous attention from the operator.

Group 5 (Layered RBC) demonstrated intermediate CP levels, which were higher than those observed in the SFRC groups (with the exception of Group 4 - Snowplow SFRC). This finding is consistent with reports indicating that layering techniques, while providing enhanced control over polymerization degree and stress, may be vulnerable to air entrapment between increments [39].

The second null hypothesis, which predicted no significant difference in DC among techniques, was only partially accepted. The DC at the top, middle and bottom regions of the different RBCs was assessed using micro-Raman spectroscopy. This technique allows for the direct detection of the amount of unreacted double bond in the resin matrix. The significant association between the restorative technique and average DC suggests that the overall restorative configuration—combining different materials and layering strategies within the cavity—has a measurable effect on polymerization efficiency. Despite the statistical model’s ability to explain approximately 18% of the DC variance, it is important to note that this moderate coefficient of determination suggests the presence of other contributing factors. These include light attenuation through the layers, filler composition, resin matrix chemistry, spatial fibre distribution, and optical continuity, among others [40]. These additional factors may also contribute to the overall conversion process within the restoration.

The DC values varied significantly with depth across all experimental groups, as confirmed by repeated measures ANOVA, which demonstrated large effect sizes for depth in each group. In Group 1 (Flow SFRC) and Group 5 (Layered RBC) the DC% followed the expected pattern of decreasing polymerization with depth, with the lowest conversion being observed at the bottom of the restoration. This is consistent with the standard loss of intensity of light as it propagates through the RBC material, a phenomenon that has been extensively documented in the context of depth of cure studies [41].

It was observed that the DC values were elevated in the lower layer in groups 2 and 3, which is not consistent with the expected results. This reversal of the polymerization gradient can be attributed to the presence of the Essentia HiFlow base layer, which was polymerized to a thickness of approximately 1 mm prior to the placement of the bulk SFRC. The low thickness and universal shade permit the transmission of light, thereby activating a high concentration of photoinitiators. Following the application and polymerization of the 3 mm EverX Flow layer, the inherent translucency of the SFRC [41] permitted the penetration of light through the material, thereby reaching and further enhancing the conversion of the base layer [42]. This is consistent with previous findings that show matching the refractive index of the fibres to that of the resin matrix increases the translucency of the RBC [43]. Additionally, the bulk polymerization of the EverX Flow increment is accompanied by the generation of exothermic heat, which can accelerate the mobility of free radicals and increase the extent of post-initiation polymerization in regions that are less directly exposed to light. Experimental studies demonstrated that elevated curing or post-cure temperatures increase DC, and that post-cure heating produces measurable DC gains [41].

The most pronounced and atypical behavior was observed in Group 4 (Snowplow SFRC), representing the snowplow technique. The middle and bottom regions exhibited very high DC values (> 90%), significantly higher than the top surface layer, with no discernible difference between the middle and bottom regions. This outcome coincided with the highest internal porosity measurement recorded among all groups. Previous study has shown that SFRCs and unreinforced monomer resins exhibit a similar DC after light curing; however, in thicker specimens, the SFRC’s ability to conduct light results in a slightly higher DC compared to the resin alone [42]. In addition, the heat capacity of the E-glass fibre contained in EverX Posterior and EverX Flow may influence polymerization kinetics by absorbing and storing part of the heat generated during light curing, potentially contributing to increased conversion values.

Although internal voids are generally expected to reduce light transmission, the present results suggest that high degree of conversion can still be achieved in these restorations despite increased porosity. However, the underlying mechanisms cannot be determined from the current experimental design and require further investigation.

In groups 1–4, the EverX Flow Dentin shade was empolyed as a covering layer for the core materials. In addition to the numerous laboratory studies demonstrating favourable surface and wear characteristics compared with many particulate-filled RBCs [44], clinical evidence also suggests that the use of flowable SFRC can be considered safe when exposed to the oral environment [45]. The DC values measured in this superficial layer were generally lower than those recorded in the middle and bottom regions (except in Group 1 - Flow SFRC). However, the limited availability of literature regarding the degree of conversion of EverX Flow Dentin shades restricts further interpretation of this finding.

The third null hypothesis, which proposed the absence of statistically significant correlations among IG, CP, DC, and application technique, was partially rejected. A statistically significant moderate positive correlation was identified between IG and DC (ρ = 0.31, *p* = 0.04), as well as between CP and DC (ρ = 0.34, *p* = 0.02). These findings suggest that higher DCs were associated with increased IG formation and CP within the tested restorative configurations. One possible explanation is that greater polymerization may be accompanied by increased polymerization shrinkage stress, potentially contributing to gap development [9, 10, 46]. The direct association between CP and DC further reinforces the notion that these parameters are controlled by distinct factors. Nonetheless, both the DC and CP are regulated, at least in part, by the dynamics of the curing process [47]. These insights reinforce the notion that the performance of RBCs should be evaluated as a multifactorial outcome influenced not only by the degree of chemical conversion but also by the physical properties of the material, cavity design, and procedural parameters during clinical application [10].

Furthermore, no statistically significant correlation was observed between IG and CP across the groups examined. This finding suggests that these two parameters, which are both indicative of the structural integrity of RBC restorations, may be governed by different mechanisms. It is evident that the primary factors influencing IG are the polymerisation shrinkage and stress, adhesion to the cavity walls, the viscosity and stiffness of the RBC, and the complexity of the cavity walls [48]. Conversely, CP is more likely to be determined by the composition and rheological behaviour of the RBC, the air entrapment dynamics during insertion and manufacturing processes, polymer homogeneity, and the amount of unreacted monomers. The present findings are consistent with those of previous micro-CT analyses, which reported that higher CP does not necessarily coincide with poorer internal adaptation [48]. These findings reinforce the concept that structural integrity of RBC restorations is multifactorial and cannot be predicted by a single parameter.

The present in vitro design allows high-resolution imaging, standardization and controlled comparison among restorative techniques but limits direct clinical extrapolation. The one-month wet storage period was chosen to permit further post-curing reactions and water-related maturation processes before analysis. As RBCs continue to polymerize after light exposure and may undergo hygroscopic changes during storage, this protocol was intended to provide measurements under more stabilized conditions than those observed immediately after curing. A critical limitation is that DC was measured at a single post-curing time point, one month after wet storage. RBCs undergo post-polymerization (“dark cure”) reactions, during which additional free-radical conversion continues after light exposure has ceased; therefore, the reported DC values likely represent stabilized or near-final conversion levels rather than the immediate post-irradiation state. Clinically, however, restorations are polymerized intraorally and may be subjected to functional loading shortly after placement, at a stage when polymerization is not yet complete. Under such conditions, ongoing conversion, stress development, and early mechanical loading may influence interfacial integrity and gap formation differently from the present experimental setting. Moreover, intraoral factors such as temperature fluctuations, humidity, and occlusal forces may further affect polymerization kinetics. In addition, the absence of thermocycling and cyclic mechanical loading may have influenced the IG and CP results, as thermal stress and fatigue loading can promote interfacial degradation, gap propagation, and structural changes within the restoration over time. Future studies incorporating aging protocols and multi-time-point DC assessments are warranted to better characterize the clinical behavior of SFRC restorations. Another methodological consideration is that DC values were compared among different resin composite materials with different chemical compositions. In the present study, the aromatic C = C peak at 1609 cm⁻¹ was used as the internal reference for EverX Posterior and Essentia HiFlo, whereas the CH₂ deformation band at 1458 cm⁻¹ was used for EverX Flow and G-aenial A’chord because these materials lack aromatic structures. Therefore, although the measurements allow evaluation of polymerization within each material and restorative configuration, direct cross-material comparison of absolute DC values should be interpreted with caution. Differences in resin matrix chemistry, filler loading, fibre content, translucency, and the selected reference peak may influence the measured DC values.

Further limitation to our study setup from a clinical point of view might be that the flowable SFRC material was utilized within the restoration without being covered by conventional packable RBC. Importantly, the scientific literature increasingly reports on the uncovered use of SFRC materials, including flowable variants, as a mechanically functional restorative material. A growing body of in vitro and emerging clinical evidence suggests that, in specific indications, these materials may be used without conventional composite coverage to enhance fracture resistance and fatigue performance [46, 49–53]. The authors would like to clarify that the aim of the presented study was not to propose an immediate clinical protocol, but rather to investigate, under controlled in vitro conditions, the material-related behavior of a flowable short fiber-reinforced RBC when applied using different techniques [5].

To summarize our findings, it can be outlined that in addition to demonstrating favourable performance with regard to IG and CP, Groups 1 and 2, representing simplified SFRC bulk flow application alone or in combination with conventional flowable RBC, exhibited satisfactory DC. These findings offer clinically relevant insights that complement a recent report demonstrating comparable fracture resistance in molar and premolar teeth restored solely with flowable micrometer-sized SFRC [54]. The data collectively suggests that achieving structural integrity and restorative performance may be more efficiently accomplished using EverX Flow, as it combines favourable mechanical behaviour with improved internal characteristics and simple application, which are paramount features in clinical scenario.

## Conclusions

Within the limitations of this in vitro study, the findings indicate that:

1. The flowable bulk-fill SFRCs provide superior cavity adaptation, reduced CP, and satisfactory DC, whether used alone or with a flowable liner. The results support that simplified bulk-fill application of flowable SFRCs is a viable alternative to conventional layering or viscous bulk-fill RBCs in deep cavities.
2. The snowplow technique, while promising in terms of DC and adaptation, introduces greater porosity if not meticulously executed.
3. The utilization of polyethylene fibre meshes warrants caution, as they disrupt interfacial continuity and promote gap formation.

## Data Availability

The data that support the findings of this study are available on request from the corresponding author. The data are not publicly available due to privacy or ethical restrictions.
